# Attention-Deficit/Hyperactivity Disorder Following Mild Traumatic Brain Injury in Children: A Retrospective Exploratory Study

**DOI:** 10.7759/cureus.96831

**Published:** 2025-11-14

**Authors:** Jimena M Astigarraga Baez, Maria E Garcia Gonzalez, Laura E Acevedo Ugarriza, Gustavo I Rivas Martinez, Emilia S Nuñez-Peña, Ryan P Kelly, Santiago Campos, Maria C Diaz, Maria D Beletanga, Alcy R Torres

**Affiliations:** 1 Pediatric Neurology, Universidad Catolica Nuestra Señora de la Asuncion, Asuncion, PRY; 2 School of Medicine, Ponce Health Sciences University, Ponce, PRI; 3 School of Medicine, Universidad Catolica Nuestra Señora de la Asuncion, Asuncion, PRY; 4 Statistics, Universidad Nacional de Asunción, San Lorenzo, PRY; 5 School of Medicine, Tecnológico de Monterrey, Monterrey, MEX; 6 College of Medicine, University of Vermont, Burlington, USA; 7 Pediatrics, Nicklaus Children's Hospital, Miami, USA; 8 Pediatrics, Boston University Chobanian and Avedisian School of Medicine, Boston, USA; 9 Biostatistics, Syneos Health, Morrisville, USA; 10 General Practice, Boston University Chobanian and Avedisian School of Medicine, Boston, USA; 11 General Practice, Boston Medical Center, Boston, USA; 12 Pediatric Neurology, Boston University Chobanian and Avedisian School of Medicine, Boston, USA

**Keywords:** adhd, adolescents, attention deficit/hyperactivity disorder, children, pediatric, traumatic brain injury

## Abstract

Introduction

This study examines whether attention-deficit/hyperactivity disorder (ADHD) can develop after traumatic brain injury in children. Overlapping acute symptoms can hinder early identification and diagnosis. We investigated whether demographic or acute clinical features are associated with the development of secondary ADHD (S-ADHD) following mild pediatric traumatic brain injury.

Materials and methods

We conducted a retrospective cohort study of patients at the Boston Medical Center Pediatric Concussion Clinic from 2010 to 2023. Of 284 screened pediatric patients with traumatic brain injury, 198 were excluded due to missing initial or last SCAT scores, pre-existing ADHD, or incomplete assessments. The final analytic sample included 86 patients, categorized into an S-ADHD group (n = 10) and a non-ADHD group (n = 76), based on a new diagnosis made by a pediatric neurologist. We compared demographics and symptom variables using descriptive statistics, the Wilcoxon rank-sum test, and Fisher’s exact test.

Results

No statistically significant differences were observed between groups in age, sex, or acute clinical symptoms (e.g., loss of consciousness, confusion, amnesia). However, children diagnosed with S-ADHD demonstrated a significantly longer follow-up duration (median 536 vs. 132 days, p < 0.001), highlighting the need for prolonged clinical monitoring in this population.

Conclusion

This exploratory study found no significant acute clinical features associated with S-ADHD development after mild traumatic brain injury. The findings underscore the importance of prolonged clinical monitoring, as S-ADHD may manifest later in life. Future research with larger cohorts is needed to identify reliable early indicators.

## Introduction

Traumatic brain injury (TBI) refers to a head injury caused by an external force event, resulting in symptoms that may appear immediately or develop over time. TBI is a leading cause of acquired neurodevelopmental impairment affecting millions of children worldwide each year. In 2019, an estimated 27.16 million new cases of TBI were reported globally, with a prevalence of 48.99 million cases [1]. The annual incidence is approximately 69 million cases, with mild TBIs accounting for 55.9 million and severe TBIs affecting 5.48 million people yearly [2]. In the United States alone, 214,110 TBI-related hospitalizations occurred in 2020, and 69,473 TBI-related deaths were recorded in 2021 [3]. While some individuals recover fully, others experience persistent neurocognitive and behavioral challenges, including an increased risk of attention-deficit/hyperactivity disorder (ADHD) [4].

ADHD is a prevalent neurodevelopmental disorder characterized by inattention, impulsivity, and hyperactivity, often leading to difficulties in academic performance, social interactions, and overall quality of life [5]. Analysis of the 2022 National Survey of Children's Health parent-report data showed that, in the United States, 11.4% of children aged 3-17 were undiagnosed with ADHD, with 10.5% having ADHD (~6.5 million). Among those with current ADHD, 58.1% experienced moderate to severe symptoms, and only about half received pharmacological treatment or behavioral therapy, at 53.6% and 44.4%, respectively. Conversely, in the previous year, one-third (30.1%) received non-specific ADHD treatment [6]. Emerging evidence suggests that children who sustain TBI are at an increased risk of developing secondary ADHD (S-ADHD), a subtype of ADHD that emerges following brain injury. Yet, this relationship remains underexplored [7-9]. Conversely, ADHD has also been identified as a potential risk factor for sustaining a TBI, highlighting a bidirectional association between these conditions [10,11].

Despite increasing recognition of the link between TBI and ADHD, the specific risk factors influencing S-ADHD development remain poorly defined. Studies suggest that factors such as injury severity, pre-existing behavioral disorders, socioeconomic status, and family environment may influence S-ADHD outcomes after TBI [12]. However, there is limited consensus on which factors play the most critical role and whether early identification of high-risk individuals can improve interventions and outcomes.

This study explores clinical, demographic, and injury-related factors associated with S-ADHD in children to identify high-risk profiles. Our focus is on mild TBI, which is a transient disturbance in brain function caused by trauma, typically self-limiting and resolving without long-term complications in most cases. Despite its prevalence, this subgroup remains underrepresented in long-term behavioral outcome research. The findings aim to enhance early detection, guide targeted interventions, and strengthen evidence-based rehabilitation. Identifying these risk factors will inform clinical decision-making in pediatric neurology. Finally, we hypothesized that acute clinical features would not significantly differ between children who developed S-ADHD and those who did not.

## Materials and methods

Study and setting

This was a retrospective, single-center cohort study conducted at Boston Medical Center's (BMC) Pediatric Neurology Specialty Clinic, Boston, Massachusetts, United States. BMC, a safety-net hospital, primarily serves uninsured or Medicaid-reliant patients. The clinic evaluated all patients referred by other healthcare providers or those who had previously received treatment in the Pediatric Neurology Specialty Clinic at BMC.

The electronic medical records (EMR) of 284 consecutive patients evaluated for TBI at the concussion clinic between January 2010 and March 2023 were extracted from the EPIC system software (Epic Systems Corporation, Verona, Wisconsin, United States). This digital database stores a patient's comprehensive health history, including diagnoses, treatments, medications, and clinical notes, thereby facilitating continuity of care and informed medical decision-making. Patients who suffered a TBI were followed throughout the study period to monitor clinical progress and outcomes.

Patients received a thorough neurological and cognitive evaluation during each appointment and were evaluated using the Sport Concussion Assessment Tool (SCAT) for the number and severity of post-concussion symptoms. The SCAT tools are developed by the Concussion in Sport Group (CISG) and are publicly available for clinical and research use under open-access terms, without charge for non-commercial purposes [13]. SCAT is a free-use standardized survey used for evaluating athletes and individuals with suspected concussion or TBI by healthcare professionals. It integrates a variety of components, including symptom checklists, cognitive screening, and neurological examination, to help clinicians identify the presence and severity of post-concussion symptoms. The SCAT is age-specific with corresponding versions adapted for children and older adolescents.

The SCAT assessment was administered at the first clinical encounter following injury, and follow-up assessments were performed as clinically indicated during recovery based on medical criteria. Patients in the S-ADHD group were those who received a new clinical diagnosis of ADHD by a board-certified pediatric neurologist during their follow-up, based on the Diagnostic and Statistical Manual of Mental Disorders, Fifth Edition (DSM-5) criteria, as documented in the electronic medical record. The physicians determined the appropriate diagnoses using specialized questionnaires, such as the Vanderbilt Parent and Teacher Questionnaires, the DSM-5 criteria, and clinical judgment based on years of experience in pediatric neurology. 

Ethics and safety

This study adhered to the ethical principles established in the Declaration of Helsinki. The Institutional Review Board of BMC approved the study protocol (H-38768), which included data management procedures. Since the research was considered low risk, the informed consent requirement was waived.

Patient selection

Eligible participants were children and adolescents aged 4-21 years who had a documented TBI and at least one SCAT (versions 2-5) completed at the initial visit and at follow-up ≥ 1 month later. The current and revised versions of SCAT utilized during the study timeframe included: SCAT 2, SCAT 3, SCAT 3 Child, SCAT 5, and SCAT 5 Child, to classify the severity of concussions. SCAT 2 used from 2008-2013, SCAT 3 from 2013-2017, and SCAT 5 from 2017-2023; together, these cover the entire study period and capture the evolution of best-practice concussion assessment [13]. SCAT 6 was not included since it was released after our data lock in late 2023 and therefore does not appear in our dataset [14]. 

The exclusion criteria for this study comprised patients who lacked a concussion diagnosis, had an existing diagnosis of ADHD before the occurrence of TBI, did not possess complete first or last SCAT data, or were not able to be followed up for a minimum of one month following the trauma.

**Figure 1:**
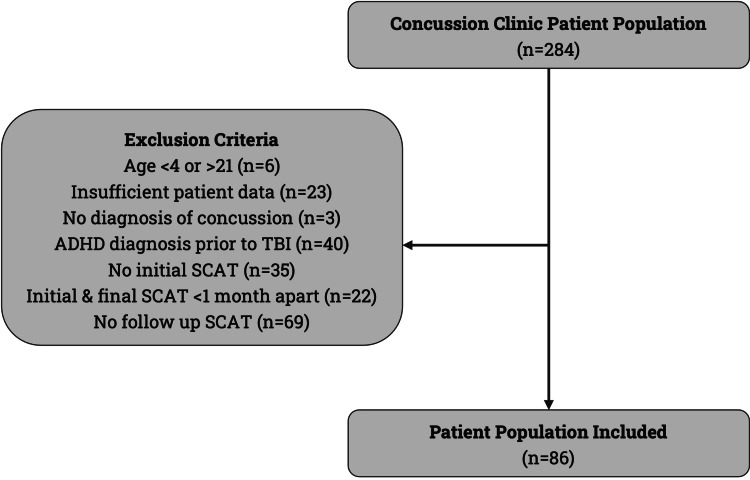
Flowchart of pediatric post-TBI patient population inclusion analyses within the BMC Concussion Clinic patient EMR from 2010 to 2023. BMC, Boston Medical Center; TBI, traumatic brain injury; EMR, electronic medical records; ADHD, Attention Deficit/Hyperactivity Disorder; SCAT, Sport Concussion Assessment Tool (versions 2–5) [13,14]

Patient selection flow based on retrospective EMR review from the Boston Medical Center Concussion Clinic is shown in Figure 1. Of the 284 pediatric patients assessed for TBI, exclusions were applied for missing initial or last SCAT scores, pre-existing ADHD, and insufficient follow-up. A total of 86 patients evaluated and treated at BMC's Pediatric Neurology Specialty Clinic were included after the application of the inclusion and exclusion criteria.

Data collection, missing data, and bias control

Data was extracted from EMRs and managed using Microsoft Excel 2021 (Microsoft Corporation, Redmond, Washington, United States). A total of 198 patients were excluded due to incomplete data (e.g., missing SCAT scores or follow-up documentation). We compared the available demographic data of included and excluded participants and found no significant differences in age or sex distribution, suggesting limited selection bias. However, the potential impact of missing data on representativeness is acknowledged as a limitation.

Statistical analysis

Our study utilized descriptive statistics to summarize the demographic and clinical characteristics of the sample, categorizing the data based on ADHD status following TBI. We reported continuous variables as means, standard deviations (SDs), medians, and interquartile ranges (IQRs). Categorical variables were presented as frequencies and percentages.

We conducted between-group comparisons to evaluate the similarities in demographic variables and explore potential associations with acute clinical symptoms. Due to the small sample size in the ADHD group, we utilized nonparametric tests. Specifically, we applied the two-tailed Wilcoxon rank-sum test [15] for continuous variables and the two-tailed Fisher's exact test [16] for categorical variables. We established a significance level of 0.05 for all analyses and performed all statistical procedures using R software (R Foundation for Statistical Computing, Vienna, Austria, https://www.R-project.org/) [17].

## Results

A total of 86 pediatric patients with TBI were included in the final analysis, of whom 10 (12%) were subsequently classified as S-ADHD. The median age at injury was lower in the S-ADHD group (13.0 years; IQR, 9.0-17.0) than in the non-ADHD group (15.5 years; IQR, 12.5-17.0), with overlapping interquartile ranges. Sex distribution was similar, with females representing 40% of the S-ADHD group and 51% of the non-ADHD group, indicating broadly comparable demographic profiles at baseline (Table 1).

**Table 1:** Descriptive analysis of demographic and clinical characteristics of pediatric patients in the two groups S-ADHD, secondary attention-deficit/hyperactivity disorder; Non-ADHD, group without attention-deficit/hyperactivity disorder post-TBI; TBI, traumatic brain injury

Variable	S-ADHD (n = 10)	Non-ADHD (n = 76)	Overall (n = 86)
Age at injury			
Mean ± SD	12.6 ± 4.2	14.8 ± 3.5	14.5 ± 3.7
Median (Q1–Q3)	13.0 (9.0–17.0)	15.5 (12.5–17.0)	15.0 (12.0–17.0)
Sex, n (%)			
Female	4 (40%)	39 (51%)	43 (50%)
Male	6 (60%)	37 (49%)	43 (50%)
Follow-up time (days)			
Mean ± SD	534 ± 349	209 ± 214	247 ± 254
Median (Q1–Q3)	536 (249–706)	132 (62–274)	151 (65–329)
Comorbidities before TBI, n (%)			
Learning disorder	0 (0%)	9 (12%)	9 (10%)
Anxiety	0 (0%)	13 (17%)	13 (15%)
Migraine history	1 (10%)	15 (20%)	16 (19%)
Play sports regularly?, n (%)			
Yes	6 (60%)	45 (59%)	51 (59%)

Follow-up duration after the injury was longer in the S-ADHD group (median 536 days; IQR, 249-706) than in the non-ADHD group (median 132 days; IQR, 62-274). This difference suggests a greater demand for ongoing clinical monitoring in patients who developed S-ADHD. While follow-up time is not a baseline variable, we can hypothesize that it may act as an indicator of the continued need for healthcare services, family involvement, and institutional support. Early identification of patients at higher risk could help target monitoring efforts, facilitate timely interventions, and optimize the use of healthcare resources (Table 1).

Pre-injury comorbidities were infrequent in the S-ADHD group: no cases of learning disorder or anxiety were recorded, and migraine history was present in one patient (10%). In the non-ADHD group, learning disorder and anxiety were reported in 12% and 17% of patients, respectively, and migraine history in 20%. Participation in regular sports prior to the injury was comparable between groups, 60% vs. 59% (Table 1).

Acute post-TBI symptoms were reported at similar proportions in both groups (Table 2). Loss of consciousness was documented in 22% of the S-ADHD group and 27% of the non-ADHD group; confusion in 22% vs. 26%; retrograde amnesia in 22% vs. 17%; and anterograde amnesia in 22% vs. 13%. Although the S-ADHD group showed slightly higher percentages for amnesia and confusion, absolute differences were minor.

**Table 2:** Descriptive analysis of symptoms at the TBI event of pediatric patients in the two groups S-ADHD, secondary attention-deficit/hyperactivity disorder; NON-ADHD, group without ADHD post-TBI; TBI, traumatic brain injury.

Variable	S-ADHD (n = 10), n (%)	NON-ADHD (n = 76), n (%)	Overall (n = 86) , n (%)
Loss of consciousness			
Yes	2 (20%)	20 (26%)	22 (26%)
No	7 (70%)	54 (71%)	61 (71%)
Not Recorded	1 (10%)	2 (2.6%)	3 (3.5%)
Confusion			
Yes	2 (20%)	19 (25%)	21 (24%)
No	7 (70%)	53 (70%)	60 (70%)
Not Recorded	1 (10%)	4 (5.3%)	5 (5.8%)
Retrograde amnesia			
Yes	2 (20%)	12 (16%)	14 (16%)
No	7 (70%)	60 (79%)	67 (78%)
Not Recorded	1 (10%)	4 (5.3%)	5 (5.8%)
Anterograde amnesia			
Yes	2 (20%)	9 (12%)	11 (13%)
No	7 (70%)	63 (83%)	70 (81%)
Not Recorded	1 (10%)	4 (5.3%)	5 (5.8%)

To assess baseline comparability, demographic characteristics were evaluated for the S-ADHD and non-ADHD groups following TBI (Table 3). The median age at injury was slightly lower in the S-ADHD group (13.0 years, Q1-Q3: 9.0-17.0) compared with the non-ADHD group (15.5 years, Q1-Q3: 12.5-17.0), but this difference was not statistically significant (p = 0.151, Wilcoxon rank-sum test). Similarly, sex distribution did not differ significantly between groups, with females representing 40% of the S-ADHD group and 51% of the non-ADHD group (p = 0.702, Fisher’s exact test). These results indicate that both groups were comparable in terms of age and sex at baseline.

**Table 3:** Comparison of age at the time of injury and sex between the two groups S-ADHD, secondary attention-deficit/hyperactivity disorder; Non-ADHD, group without ADHD post-TBI.

Variable	S-ADHD (n = 10)	Non-ADHD (n = 76)	Test Name	Test Statistic	p-value
Age at injury (years)					
Mean ± SD	12.6 ± 4.2	14.8 ± 3.5	Wilcoxon rank-sum	W = 329.0	0.151
Median (Q1–Q3)	13.0 (9.0–17.0)	15.5 (12.5–17.0)			0.151
Sex, n (%)					
Female	4 (40%)	39 (51%)	Fisher’s exact test	OR = 0.63	0.702
Male	6 (60%)	37 (49%)			0.702

The study assessed the presence of acute clinical symptoms at the time of injury and their potential association with a subsequent diagnosis of S-ADHD, as well as differences in follow-up duration between groups. As presented in Table 4, no statistically significant differences were observed for loss of consciousness (p > 0.9), confusion (p > 0.9), retrograde amnesia (p = 0.6), or anterograde amnesia (p = 0.6). Although the S-ADHD group exhibited slightly higher proportions of amnesia and confusion compared with the non-ADHD group, these differences were minor and did not reach statistical significance. This suggests that acute symptoms may not help predict subsequent ADHD. In contrast, follow-up duration was significantly longer in the S-ADHD group (median 536 days, Q1-Q3: 249-706) than in the non-ADHD group (median 132 days, Q1-Q3: 62-274; p = 0.01). Moreover, individuals who develop S-ADHD tend to require longer clinical monitoring as an outcome. 

**Table 4:** Comparison of acute post-TBI symptoms and follow-up time Note: Data are missing for certain variables and the sample size (n) for each variable is given to accurately reflect the number of patients with available data in each analysis S-ADHD, secondary attention-deficit/hyperactivity disorder; NON-ADHD, group without ADHD post-TBI; TBI, traumatic brain injury.

Variable	Total	S-ADHD	NON-ADHD	Test Name	Test Statistic	p-value
Acute post-TBI symptoms						
Loss of consciousness	83	n = 9	n = 74	Fisher’s exact test	OR = 0.77	>0.9
		2 (22%)	20 (27%)			
Confusion	81	n = 9	n = 72	Fisher’s exact test	OR = 0.79	>0.9
		2 (22%)	19 (26%)			
Retrograde amnesia	81	n = 9	n = 72	Fisher’s exact test	OR = 1.42	0.6
		2 (22%)	12 (17%)			
Anterograde amnesia	81	n = 9	n = 72	Fisher’s exact test	OR = 2.04	0.6
		2 (22%)	9 (13%)			
Follow-up time (days)						
Median (Q1–Q3)	86	n = 10	n = 76	Wilcoxon rank-sum	W = 626	0.01
		536 (249–706)	132 (62–274)			

## Discussion

This exploratory study aimed to assess whether demographic and acute clinical features could help predict the development of S-ADHD following pediatric TBI. Given the small number of S-ADHD cases (n = 10), the results should be interpreted as hypothesis-generating. Our findings revealed no statistically significant differences in age, sex, or acute symptoms (including loss of consciousness, confusion, anterograde and retrograde amnesia) between children who developed S-ADHD and those who did not. These results suggest that early clinical presentations may not reliably identify children at elevated risk for S-ADHD after TBI.

Our data are consistent with prior research establishing a relationship between pediatric TBI and increased S-ADHD risk [4,18-20]. A meta-analysis reported on the correlation between TBI and S-ADHD, demonstrating a robust association, most predominantly in patients diagnosed with moderate-to-severe cranial injuries, and emphasized the importance of long-term monitoring for neurobehavioral sequelae in the pediatric population [4]. Additionally, a population-based study reported that children with TBI had a 32% higher risk of developing ADHD compared to their non-TBI counterparts [18]. These studies focused on a predominantly moderate-to-severe TBI presentation, whereas our study’s population consisted of patients with diagnosed mild TBI. Moreover, recent literature has begun to explore the impact of mild TBI on general neurocognitive functioning in pediatric populations, and how the symptomatology may present further down the line [20,21]. Our study expands the line of inquiry to examine a predominantly mild TBI cohort, emphasizing that even in the absence of severe injury, ADHD specifically may manifest over time in a subset of patients. The outcomes of mild TBI, which constitute most pediatric cases, remain poorly understood within the literature.

A pertinent finding in our analysis was the significantly prolonged follow-up duration between diagnosis and the last follow-up appointment in patients who developed S-ADHD. This extended interval with clinical services may reflect the delayed appearance of cognitive and behavioral symptomatology, reinforcing the importance of longitudinal observation of this population [22]. While our findings suggest that children with S-ADHD had longer and more frequent follow-up visits, this likely reflects the persistence or delayed emergence of symptoms prompting reevaluation, rather than serving as a predictive marker of S-ADHD. Although the exact timing of S-ADHD diagnosis relative to injury could not be determined, all diagnoses were documented during follow-up visits after the initial mTBI event. This finding further underscores the importance of ongoing clinical surveillance to identify delayed behavioral manifestations. These data points further suggest that symptom onset may be insidious and not identifiable in the immediate aftermath of the injury [23]. Other studies included significantly longer follow-up periods, allowing prolonged observation of the patient population [4,18,24], whereas the nature of this retrospective analysis lends itself to limitations of clinician intervention and patient compliance with follow-up visits.

A significant strength of this study is its focus on a relatively underrepresented population, children with mild TBI, who are often excluded from long-term behavioral outcome studies even though these comprise the majority of TBI cases [25,26]. Additionally, the use of structured concussion assessments and systematic EMR reviews enhances the consistency of data collection.

Due to the retrospective nature of data collection, this study has certain limitations. The large proportion of excluded patients may affect the generalizability of our findings and limit external validity. Furthermore, the small number of S-ADHD cases reduces statistical power. As a retrospective single-center study, findings may also be influenced by referral patterns, non-adherence to follow-up, and missing data. The lack of standardized follow-up intervals may have introduced detection bias, as increased clinical encounters could have raised the likelihood of identifying ADHD symptoms. Despite these limitations, our findings underscore the importance of systematic, long-term monitoring following pediatric TBI, even in mild cases, to better detect and address emerging behavioral disorders such as ADHD.

Despite limitations in sample size and study power, our findings reveal a statistically significant trend of prolonged follow-up in children diagnosed with S-ADHD after mild TBI. This underscores the importance of continued investigation into this often-overlooked subgroup. Our study contributes to the limited literature on mild TBI in pediatric populations and highlights the potential for long-term neurodevelopmental consequences that may not be immediately apparent. Future prospective studies with larger samples and standardized follow-up intervals are needed to clarify early predictors of S-ADHD and to guide targeted early intervention strategies.

## Conclusions

In this cohort of pediatric patients with mild TBI, no statistically significant differences were found in acute clinical symptoms between those who developed S-ADHD and those who did not. Early post-injury features such as loss of consciousness, confusion, or amnesia did not appear to identify children at higher risk. However, children diagnosed with S-ADHD had significantly longer follow-up durations, which likely reflect delayed recognition of behavioral and cognitive changes rather than serving as a predictive marker of S-ADHD. The extended follow-up also underscores the potential healthcare and family burden associated with the diagnosis.

These findings highlight the importance of maintaining long-term follow-up for all pediatric TBI patients, even when initial symptoms appear mild, to facilitate early detection and management of delayed-onset neurobehavioral outcomes such as ADHD. Although limited by sample size and single-center design, this study contributes new evidence on mild TBI, an underrepresented but prevalent subgroup, and emphasizes the need for larger, prospective studies to identify early predictors and optimize long-term care strategies.
